# The global and national energy systems techno-economic (GNESTE) database: Cost and performance data for electricity generation and storage technologies

**DOI:** 10.1016/j.dib.2024.110669

**Published:** 2024-06-25

**Authors:** Luke Hatton, Nathan Johnson, Lara Dixon, Bosi Mosongo, Savanha De Kock, Andrew Marquard, Mark Howells, Iain Staffell

**Affiliations:** a Chemical Engineering, Imperial College London, London, United Kingdom; b Centre for Environmental Policy, Imperial College London, London, United Kingdom; c Climate Compatible Growth, STEER Centre, Geography, Loughborough University, Leicestershire, United Kingdom; d Energy Systems Research Group, University of Cape Town, Cape Town, South Africa

**Keywords:** Electricity generation, Electricity storage, Capital costs, Operating costs, Financing costs, Efficiency

## Abstract

Power sector and energy systems models are widely used to explore the impacts of demographic, socio-economic or policy changes on the cost and emissions of electricity generation. Technology cost and performance data are essential inputs to such models. Despite the ubiquity and importance of these parameters, there is no standardised database which collates the variety of values from across the literature, so modellers must collect them independently each time they populate or update model inputs, leading to duplicated efforts and inconsistencies which can profoundly influence model results. Technology cost and performance varies between countries, regions and over time, meaning that data must be country- or region-specific and frequently updated. Values also vary widely between sources, so obtaining a broad consensus view is critical. Here, we present a database which collates historical, current, and future cost and performance data and assumptions for the six most prominent electricity generation technologies; coal, gas, hydroelectric, nuclear, solar photovoltaic (PV) and wind power, which together accounted for over 92 % of installed generation capacity in 2022. In addition, we provide the same data for utility-scale battery energy storage systems (BESS), regarded as critical to the integration of variable renewables such as wind and solar PV. The data are global in scope but with regional and national specificity, covers the years 2015 through to 2050, and span 5518 datapoints from 56 sources. The database enables modellers to select and justify model input data and provides a benchmark for comparing assumptions and projections to other sources across the literature to validate model inputs and outputs. It is designed to be easily updated with new sources of data, ensuring its utility, comprehensiveness, and broad applicability in future.

Specifications Table

**Table:** 

Subject	Energy
Specific subject area	Energy system modelling
Type of data	Table, Figure, .xlsx file, .csv file Raw, Processed
Data collection	Data were collected and harmonised from websites, reports, academic articles and databases of international organisations and national entities through a comprehensive literature review.
Data source location	Global with regional and national specificity.
Data accessibility	Repository name: Zenodo Data identification number: 10.5281/zenodo.11065566. Direct URL to data: https://zenodo.org/records/11065566 Repository name: GitHub Direct URL to data: https://github.com/iain-staffell/GNESTE

## 1 Value of the Data

• The database is both comprehensive and open-source. It can be used to select and justify model inputs helping to overcome issues with data inaccessibility which are a considerable barrier to developing and calibrating energy and power systems models, particularly in developing nations.
• The spatial and temporal coverage and country-level breakdown make the database applicable to a wide range of models covering different geographic regions and time horizons.
• All recordings use a consistent structure, units, and currencies allowing different sources to be compared quickly, and provide a means of validating both model inputs and outputs.
• The parameters recorded in the database are of broad utility across many types of model and are therefore in high demand among the energy modelling community.
• Example applications include calculating levelized costs of electricity generation, finding cost-effective decarbonisation pathways, and optimising power sector investment and operation.

## 2 Background

Global temperatures are rising which is having an unprecedented impact on the global energy system and human society [1]. Accelerating global decarbonisation efforts is essential if the world is to limit further warming [2]. Power sector and energy systems models are widely used to explore the impact of demographic, socio-economic and policy changes on the cost and emissions of electricity generation. Technology cost and performance data are essential inputs to these models. Cost and performance vary by region and over time, meaning that data must be region-specific and constantly updated, and are typically represented using average values based on available data, so they also vary by source. Despite their ubiquity and importance, there is no standardised database which collates technology cost and performance estimates from across the literature, so modellers must collect them independently each time they populate or update model inputs, leading to duplicated efforts and inconsistencies between studies. There are many influential sources which cover multiple technologies from the IEA [3,4], IRENA [5], EIA [6], NREL [7], CSIRO [8], Danish Energy Agency [9], DESNZ [10], and Lazard [11], among others. However, these exist in a variety of formats, using different units, customs and currencies, which we have harmonised in the GNESTE database.

Here, we present an open-access database of cost and performance data from the open literature covering seven key power generation and storage technologies:

• Coal and natural gas: which supply 35 % and 23 % of global electricity respectively, but must both be rapidly phased down to meet global decarbonisation objectives [12]. Fossil-fuelled plants are considered both as conventional unabated plants, and equipped with carbon capture and storage (CCS).
• Hydroelectric and nuclear power: which are the two largest sources of low-carbon energy, supplying 15 % and 9 % of global electricity respectively [12].
• Solar PV: which supplies 5 % of global electricity, and has grown ten-fold in the decade to 2022 [12].
• Wind energy: which supplies 7 % of global electricity, and has grown three-fold in the decade to 2022 [12].
• Battery energy storage systems (BESS): which are the fastest growing form of power system flexibility and will be critical to integrating large shares of variable renewable energy [13].

The database was assembled for use with the OSeMOSYS framework [14], but it is equally applicable to other energy and power system models. It aims to provide an accessible, useable and extendable resource for modellers, policymakers, and other stakeholders worldwide. This database streamlines the process of model setup and calibration, reducing the need to duplicate efforts when comparing or validating model inputs and outputs.

## 3 Data Description

### 3.1 Definition of units

The database covers nine parameters needed to model the generating costs of electricity generation and storage which are defined in Table 1. All cost parameters are given in 2023 US Dollars.

**Table 1:** Description of the cost and performance parameters collated in the database.

Parameter	Code	Unit	Description
Capital Cost	CAPEX	USD/kW	Overnight cost of building the plant (excluding cost escalation and interest during construction) normalised by the rated capacity of the plant.
Fixed O&M	OPEX_F	USD/kW/yr	Operation and maintenance costs which are a function of a plant's capacity, such as for labour and insurance.
Variable O&M	OPEX_V	USD/MWh	Operation and maintenance costs which are a function of operating hours, excluding fuel inputs.
Total O&M	OPEX_T	USD/kW/yr or USD/MWh	The sum of variable and fixed operating costs, excluding fuel inputs.
Fuel Price	FUEL_PRICE	USD/MWh	Cost of the fuel consumed, relative to the primary energy input (not including plant efficiency).
Efficiency	EFFICIENCY	%	Share of the net calorific value of a plant's fuel that is converted into electricity, net of plant self-consumption.
Construction Time	BUILDTIME	Years	Time to complete the project from physical installation through to electricity generation. Excludes pre-construction stages such as planning and permitting.
Lifetime	LIFETIME	Years	Time a plant is expected to operate, excluding end-of-life extensions, over which the capital cost is amortised.
Cost of Capital	WACC	%	Weighted average cost of capital used for discounting, in real terms, reflecting the split between equity and debt investments.

For BESS and hydroelectric power (specifically pumped hydro storage), the efficiency variable instead refers to the round-trip efficiency of charging and discharging, net of plant self-consumption. Costs for BESS can be measured relative to total energy storage capacity instead of maximum power output, so entries for Capital Cost are measured in both USD/kW and USD/kWh, while Fixed O&M and Total O&M are measured in both USD/kW/yr and USD/kWh/yr depending on which metric was used by each source. It is possible to convert between these using the Energy:Power Ratio of the storage system (kWh/kW, or simply hours), which is given in the database.

The GNESTE database includes historical data from 2015 to 2023 and projections for the years 2024, 2025, 2030, 2040 and 2050. Data were collected from reports, academic articles, webpages and databases of national and international organizations. In adherence to U4RIA1 guidelines, the data are retrievable, reusable, repeatable, reconstructable, interoperable, and auditable.

### 3.2 Definition of technologies

The GNESTE database covers seven technologies which are collectively divided into *33* categories, which are presented in Table 2. Full definitions of each category can be found in the Metadata of the GNESTE database.

**Table 2:** Summary of the categories of each technology collated in the database.

Technology	Category	Technology	Category
Coal	Coal	Natural Gas	Boiler
	Coal – Sub-critical		Engine
	Coal – Super-critical		Open-Cycle Gas Turbine (OCGT)
	Coal – Super-critical with CCS		Combined-Cycle Gas Turbine (CCGT)
	Coal – Ultra-supercritical		CCGT with CCS
	Coal – Ultra-supercritical with CCS	Solar	Fixed
	Lignite – Sub-critical		Single Axis Tracking
	Lignite – Super-critical		Double Axis Tracking
	Lignite – Ultra-supercritical		Rooftop Systems
	Lignite – Super-critical with CCS	Wind	Onshore
Hydroelectric	Run of River		Offshore
	Reservoir		Floating Offshore
	Lock	Batteries	Lead-acid
	Pumped Hydro Storage		Lithium-ion
Nuclear	Gen III Designs		Sodium Sulphur
	Gen III+ Designs		Vanadium Redox Flow
	Small Modular Reactors		

### 3.3 Summary of values for coal power

Table 3 compiles the sources used for each variable and how many values were collated.

**Table 3:** The number of datapoints collected and sources used for each parameter in the database for coal power.

Parameter	Number of datapoints	Sources
Capital cost	174	[3,4,8,11,[15], [16], [17], [18]]
Variable operating costs	27	[11,15,17]
Fixed operating costs	27	[11,15,17]
Total operating costs	22	[3,19]
Fuel price	72	[3,11,[19], [20], [21]]
Cost of capital	No data found	
Efficiency	24	[15,22,23]
Construction time	2	[11]
Operational lifetime	5	[11,15]

**Fig. 1:**
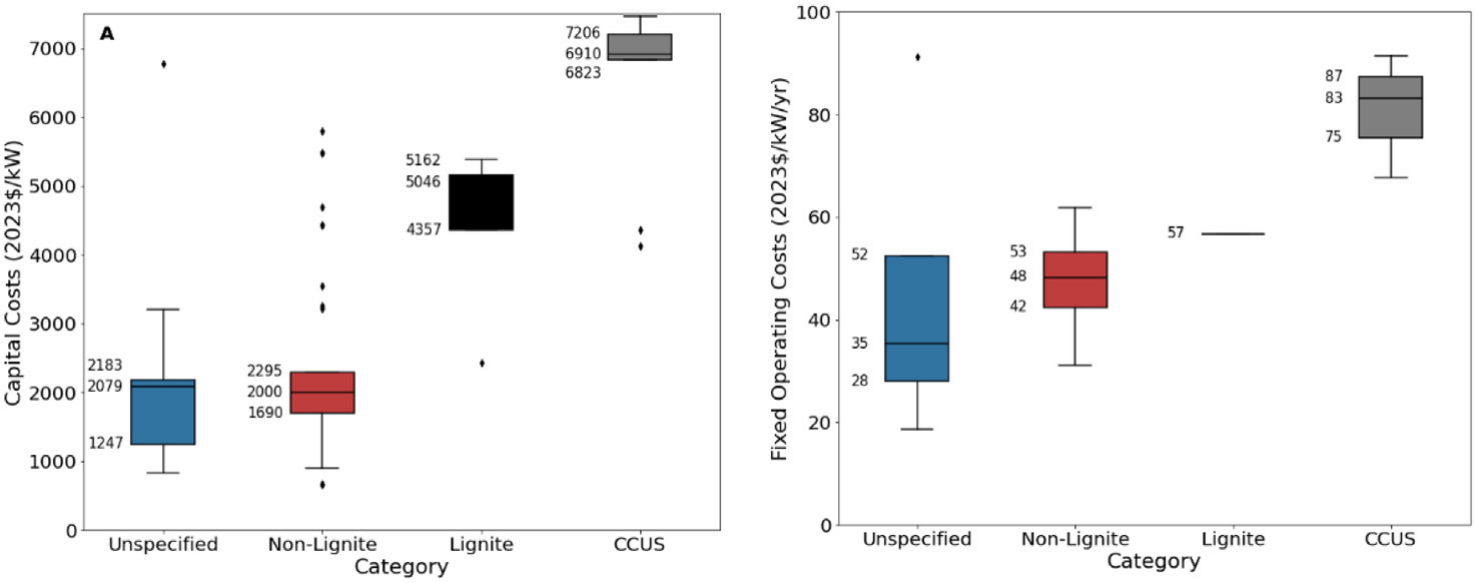
Range of a) capital costs and b) fixed operating costs for coal-fired power stations, by category in 2020 to 2024. The lower and upper bounds of each shaded box represent the lower and upper quartile of the data (25th and 75th percentile respectively), with the central line representing the median. Whiskers represent the range of values falling within 1.5 times the inter-quartile range, and outliers are shown with diamonds. The 25th, 50th and 75th percentile values are written to the left of each bar.

**Fig. 2:**
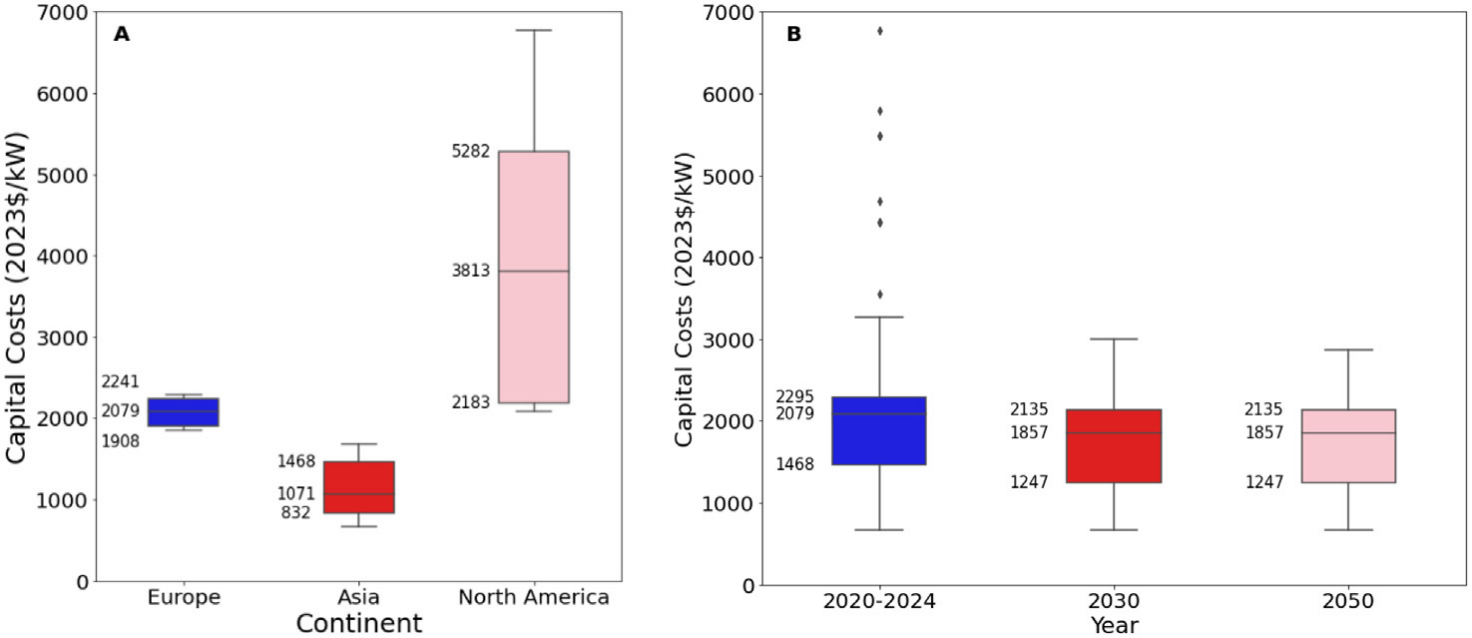
Range of operating costs for coal-fired power stations in a) 2020 to 2024 for each world region, where available, and b) global projections for 2030 and 2050.

Fig. 1, Fig. 2 present an excerpt of the values exhibited across the literature for key parameters for the recent period (2020 to 2024), alongside projections for the near (2030) and far future (2050). Fig. 1 presents the range of fixed operating and capital costs for different categories of coal-fired power stations, whilst Fig. 2 presents values of capital costs for coal (exc. Lignite) (an aggregate of ‘Coal’, ‘Coal – Supercritical’, ‘Coal – Ultrasupercritical’, and ‘Unspecified’), both regional and projected.

The median values for the recent period (2020 to 2024), aggregated across all world regions and all technology sub-types was 2235 USD/kW for capex, 53.5 USD/kW/yr for fixed O&M, 5.3 USD/MWh for variable O&M, 6.4 USD/MWh for fuel price, 39.5 % for efficiency, 5.25 years for construction time, and 40 years for operating life. There were no data for total O&M within these years.

### 3.4 Summary of values for gas power

Table 4 compiles the sources used for each variable and how many values were collated.

**Table 4:** The number of datapoints collected and sources used for each parameter in the database for gas power.

Parameter	Number of datapoints	Sources
Capital cost	263	[3,4,[8], [9], [10], [11],[15], [16], [17], [18], [19],^24^]
Variable operating costs	47	[[9], [10], [11],^15^,^17^,^18^,^24^]
Fixed operating costs	59	[[9], [10], [11],^15^,[17], [18], [19],^24^]
Total operating costs	26	[3]
Fuel price	36	[3,11,19]
Cost of capital	6	[10]
Efficiency	64	[9,10,15,[22], [23], [24]]
Construction time	51	[[9], [10], [11],^17^]
Operational lifetime	68	[[9], [10], [11],^15^,^19^,^24^]

**Fig. 3:**
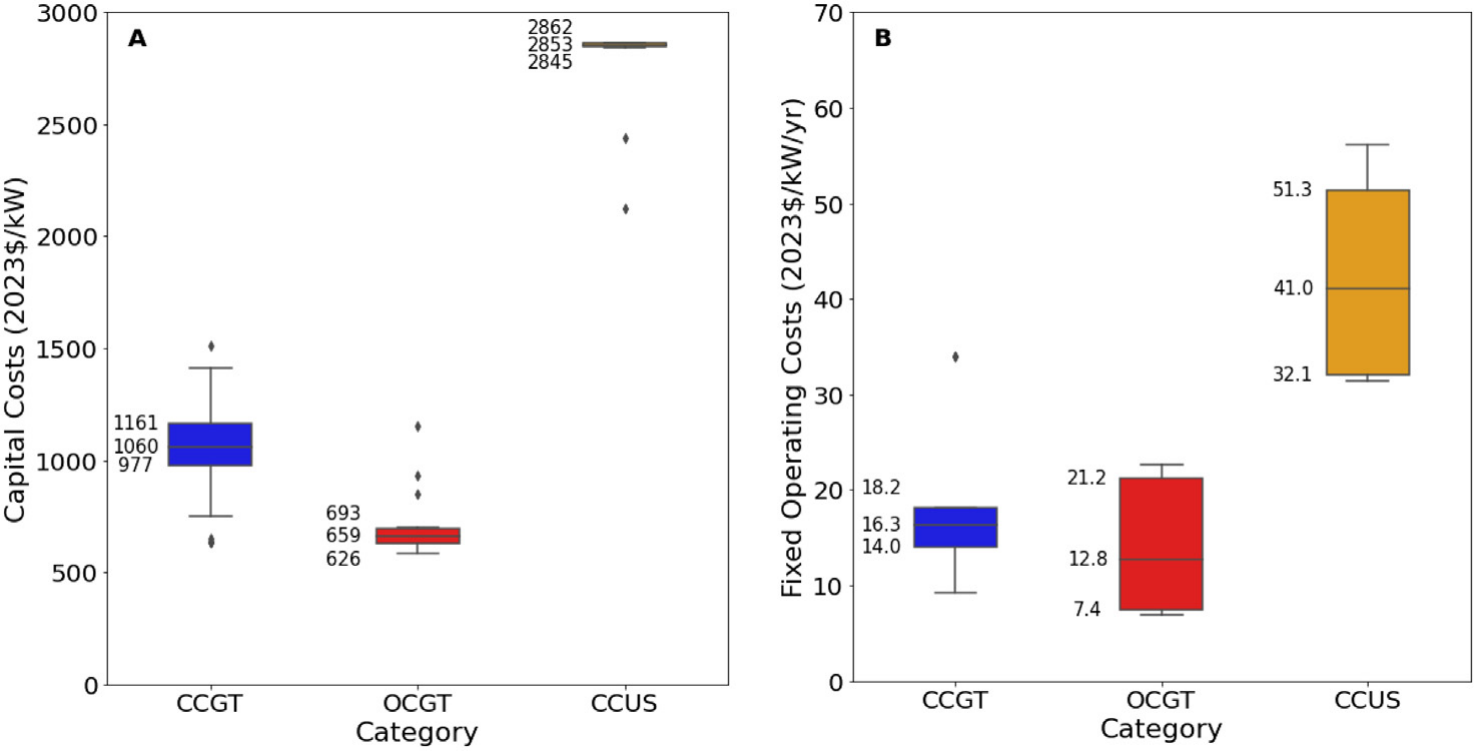
Range of a) capital costs and b) fixed operating costs for gas-fired power stations in 2020 to 2024, by category.

**Fig. 4:**
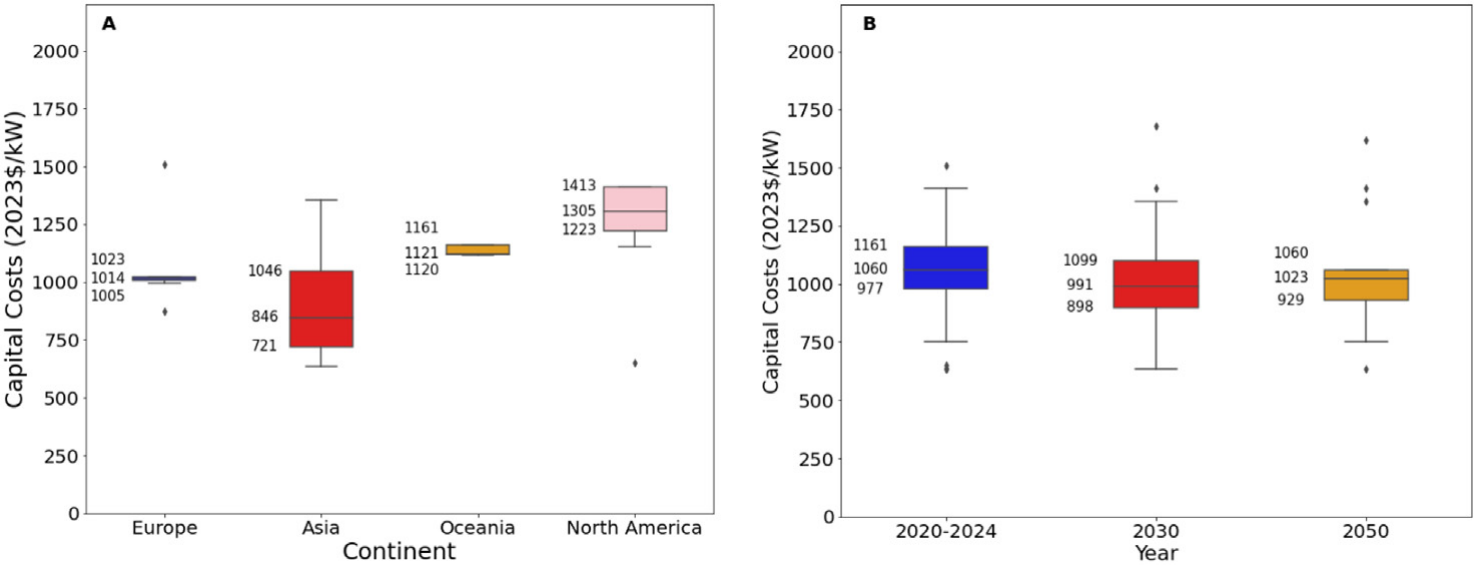
Range of capital costs for gas-fired power stations in a) 2020 to 2024 for each world region, where available, and b) global projections for 2030 and 2050.

Fig. 3, Fig. 4 present an excerpt of the values exhibited across the literature for key parameters for the recent period (2020 to 2024), alongside projections for the near future (2030) and far future (2050). Fig. 3 presents the range of fixed operating and capital costs for the different categories of gas-fired power stations for 2020–2024 whilst Fig. 4 presents values of capital costs for power plants using closed-cycle gas turbine (the ‘CCGT’ category), both regional and projected.

The median values for the recent period (2020 to 2024), aggregated across all world regions and all technology sub-types was 1040 USD/kW for capex, 17 USD/kW/yr for fixed O&M, 4.0 USD/MWh for variable O&M, 5.0 USD/MWh for total O&M, 11.8 USD/MWh for fuel price, 45 % for efficiency, 1.5 years for construction time, and 25 years for operating life.

### 3.5 Summary of values for hydroelectric power

Table 5 compiles the sources used for each variable and how many values were collated.

**Table 5:** The number of datapoints collected and sources used for each parameter in the database for hydroelectric power.

Parameter	Number of datapoints	Sources
Capital cost	594	[3,[5], [6], [7],^10^,^15^,^16^,^18^,[25], [26], [27]]
Variable operating costs	15	[10,15,18]
Fixed operating costs	303	[7,10,15,26]
Total operating costs	67	[3,6,18,27]
Fuel price	Not used	
Cost of capital	6	[10]
Efficiency	No data found	
Construction time	10	[6,7,10,18,26]
Operational lifetime	16	[6,10,15,26]

**Fig. 5:**
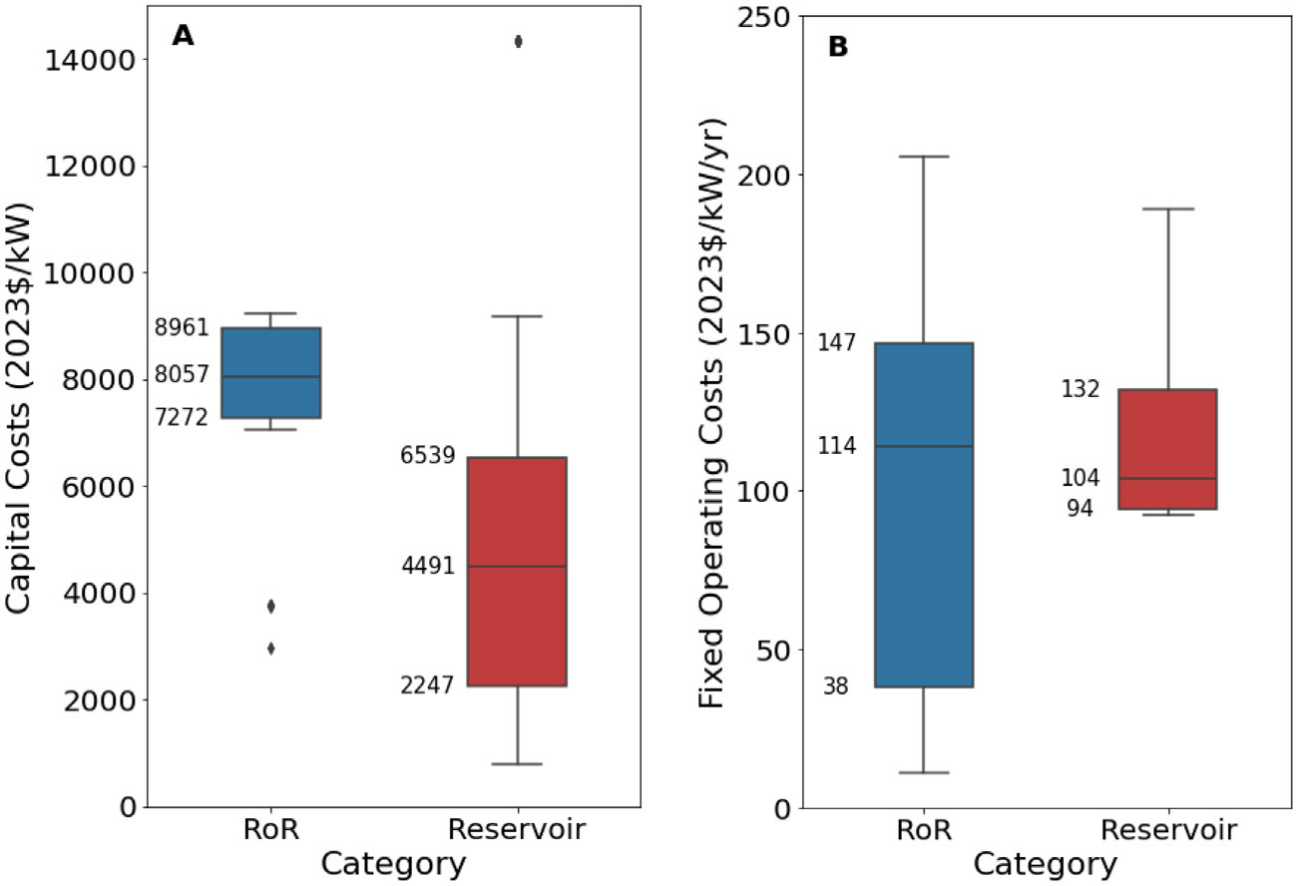
Range of a) capital costs and b) fixed operating costs for hydroelectric power stations in 2020 to 2024, for reservoir and run-of-river (RoR).

**Fig. 6:**
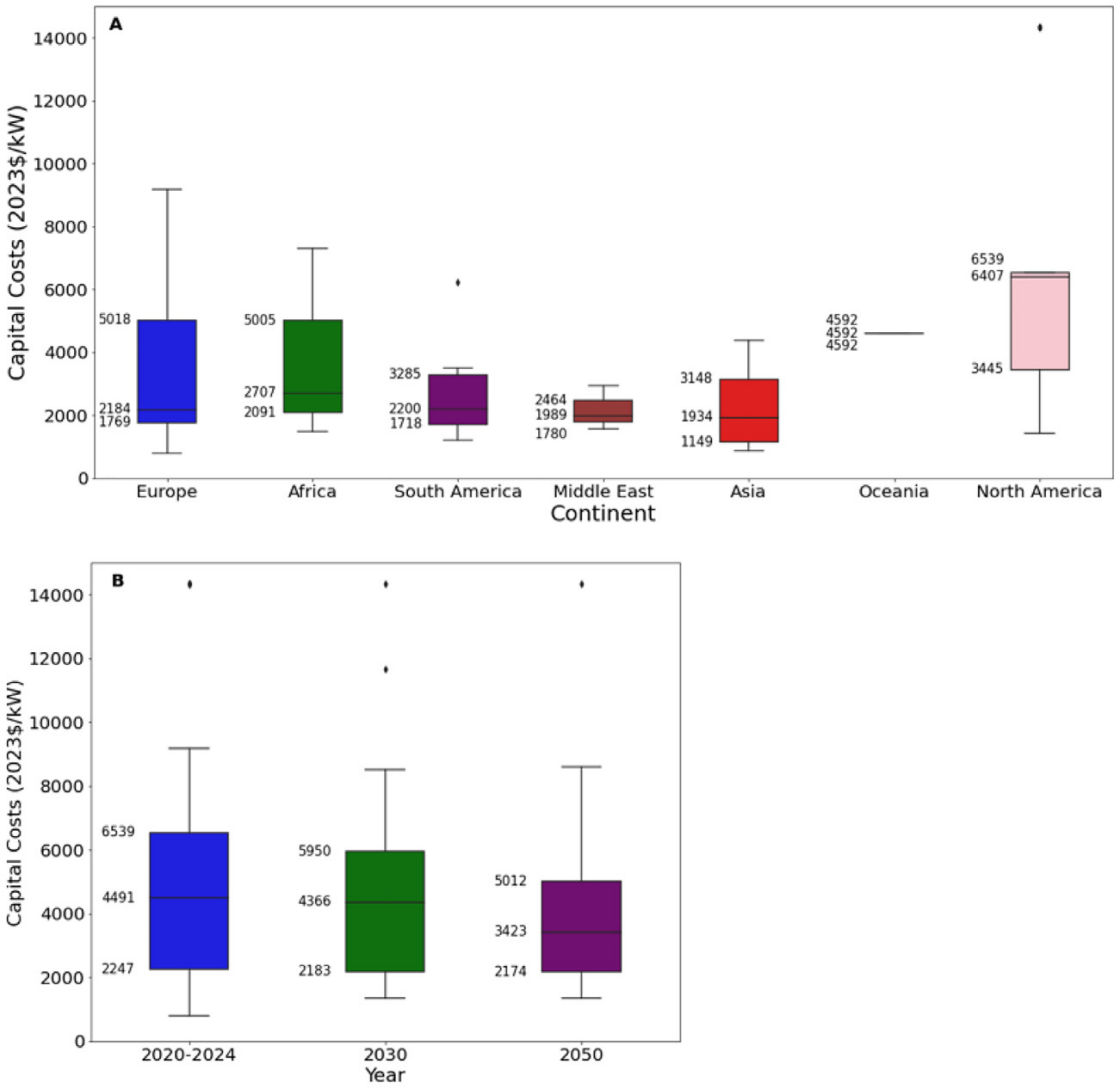
Range of operating costs for hydroelectric power stations in a) 2020 to 2024 for each world region, where available, and b) global projections for 2030 and 2050.

Fig. 5, Fig. 6 present an excerpt of the values exhibited across the literature for key parameters for the recent period (2020 to 2024), alongside projections for the near future (2030) and far future (2050). Fig. 5 presents the range of fixed operating and capital costs for the reservoir and run of river (RoR) categories of hydroelectric power stations for 2020 to 2024 whilst Fig. 6 presents values of capital costs for reservoir-based hydroelectric power stations, both regional and projected.

The median values for the recent period (2020 to 2024), aggregated across all world regions and all technology sub-types was 6407 USD/kW for capex, 92.3 USD/kW/yr for fixed O&M, 0.4 USD/MWh for variable O&M, 22.0 USD/MWh for total O&M, 4 years for construction time, and 50 years for operating life.

### 3.6 Summary of values for nuclear power

Table 6 compiles the sources used for each variable and how many values were collated.

**Table 6:** The number of datapoints collected and sources used for each parameter in the database for nuclear power.

Parameter	Number of datapoints	Sources
Capital cost	243	[3,4,[6], [7], [8],^11^,^15^,^16^,^18^,^26^,[28], [29], [30], [31]]
Variable operating costs	94	[3,6,7,11,15,17,18,26,30]
Fixed operating costs	90	[3,6,7,11,15,18,26,28,30]
Total operating costs	68	[3,4,29]
Fuel price	66	[7,11,19,23,29,30]
Cost of capital	No data found	
Efficiency	6	[15,22,30]
Construction time	22	[3,6,11,26,28,30,32]
Operational lifetime	15	[3,6,11,15,26,28,30]

**Fig. 7:**
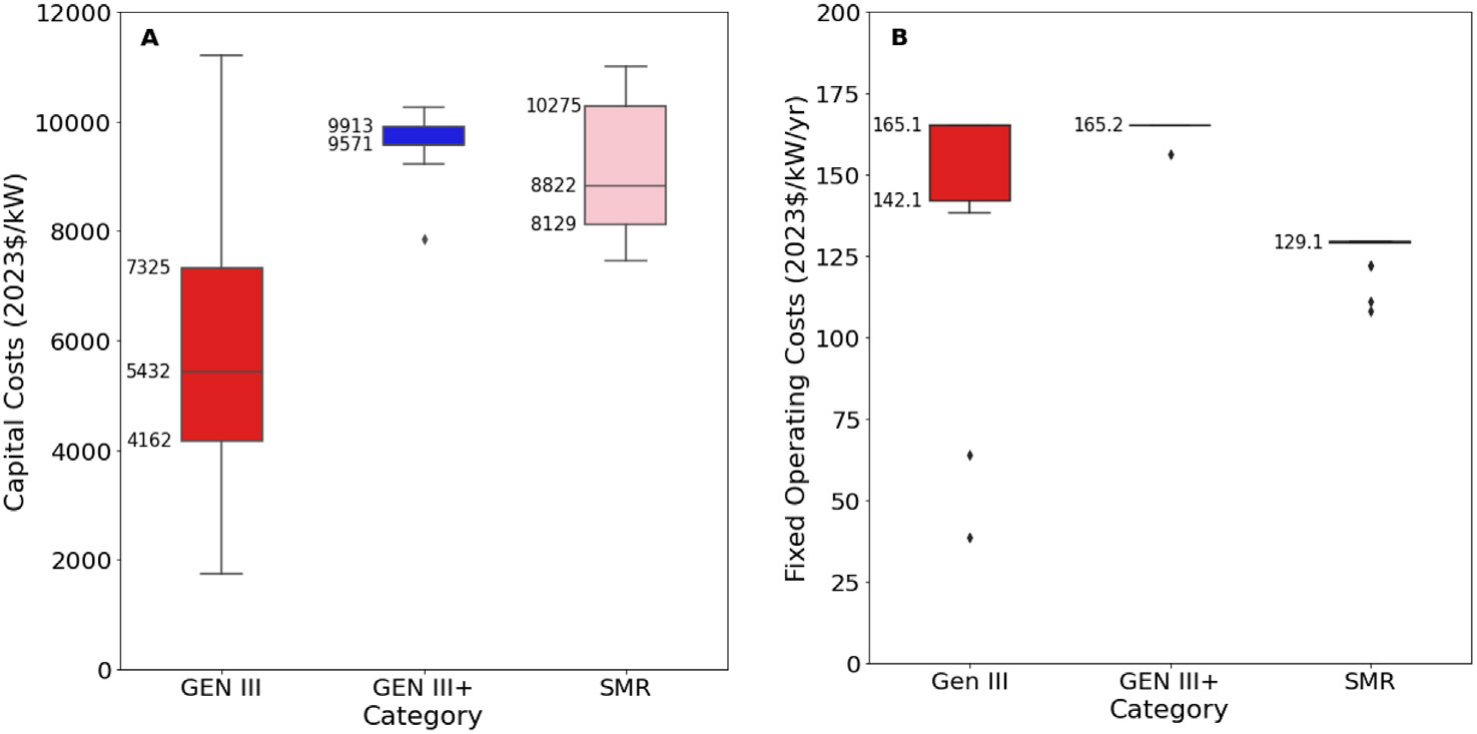
Range of a) capital costs and b) fixed operating costs for nuclear power stations in 2020 to 2024, for each category.

**Fig. 8:**
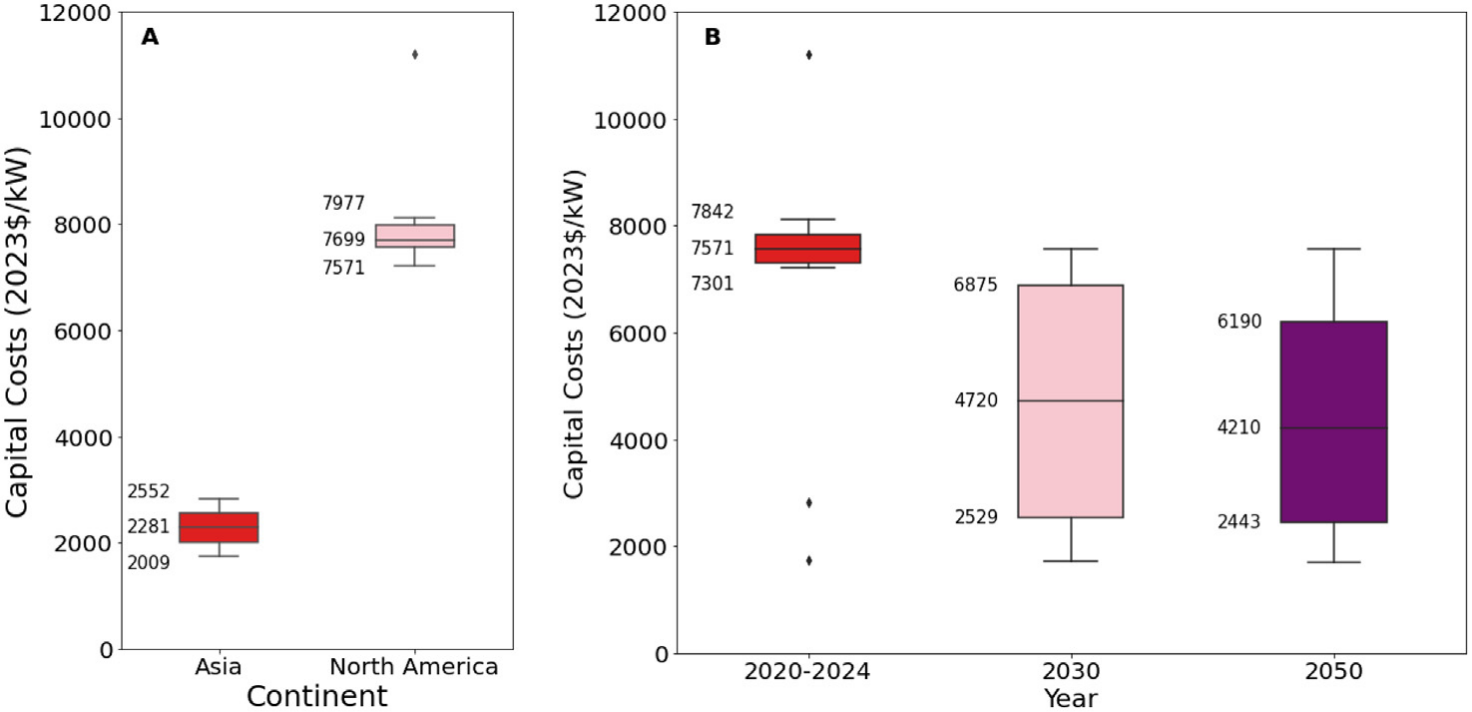
Range of operating costs for nuclear power stations in a) 2020 to 2024 for each world region, where available, and b) global projections for 2030 and 2050.

Fig. 7, Fig. 8 present an excerpt of the values exhibited across the literature for key parameter in each world region for the recent period (2020 to 2024), alongside projections for the near future (2030) and far future (2050). Fig. 7 presents the range of fixed operating and capital costs for different categories of nuclear power for 2020 to 2024, whilst 8 presents values of capital costs for Generation III technologies (the aggregate of ‘PWR’, ‘LWR’, ‘PHWR’ ‘VVER’), both regional and projected.

The median values for the recent period (2020 to 2024), aggregated across all world regions and all technology sub-types was 7350 USD/kW for capex, 131.5 USD/kW/yr for fixed O&M, 3.1 USD/MWh for variable O&M, 31.2 USD/MWh for total O&M, 6.90 USD/MWh for fuel price, 38 % for efficiency, 7.4 years for construction time, and 40 years for operating life.

### 3.7 Summary of values for solar PV

Table 7 compiles the sources used for each variable and how many values were collated.

**Table 7:** The number of datapoints collected and sources used for each parameter in the database for solar PV power.

Parameter	Number of datapoints	Sources
Capital cost	333	[[3], [4], [5], [6], [7], [8], [9], [10], [11],[15], [16], [17], [18], [19],[25], [26], [27], [28],[33], [34], [35], [36], [37]]
Variable operating costs	9	[10]
Fixed operating costs	43	[10,27,28]
Total operating costs	121	[[3], [4], [5], [6], [7],^11^,^15^,[17], [18], [19],^26^,^33^,^35^,^38^]
Fuel price	Not used	
Cost of capital	199	[10,25,35,[39], [40], [41], [42]]
Efficiency	Not used	
Construction time	22	[9,10,28,33,43]
Operational lifetime	26	[3,7,9,10,15,19,25,27,28,34,38,44]

**Fig. 9:**
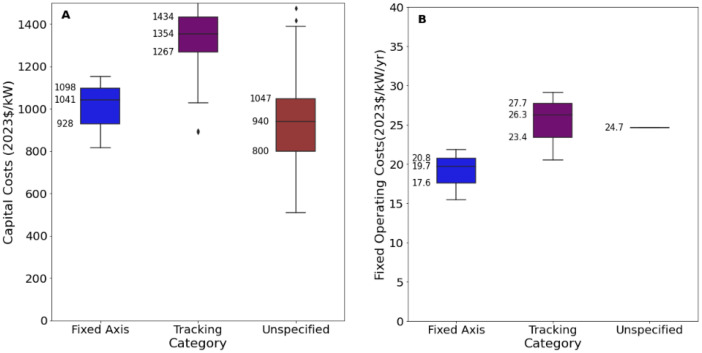
Range of a) capital costs and b) fixed operating costs in 2020 to 2024 for solar PV generation, by category.

**Fig. 10:**
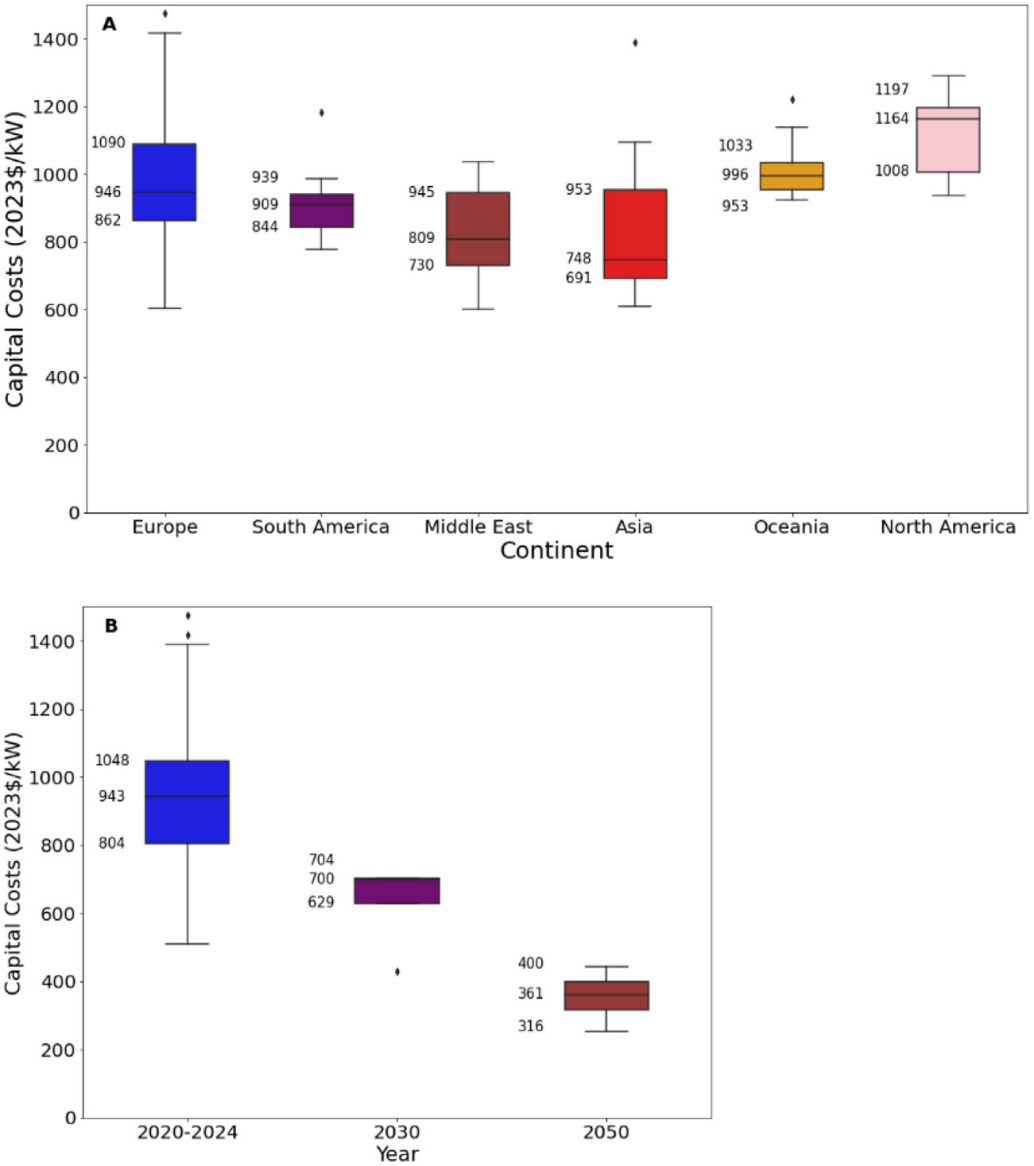
Range of capital costs for solar farms in a) 2020 to 2024 for each world region, where available, and b) global projections for 2030 and 2050.

**Fig. 11:**
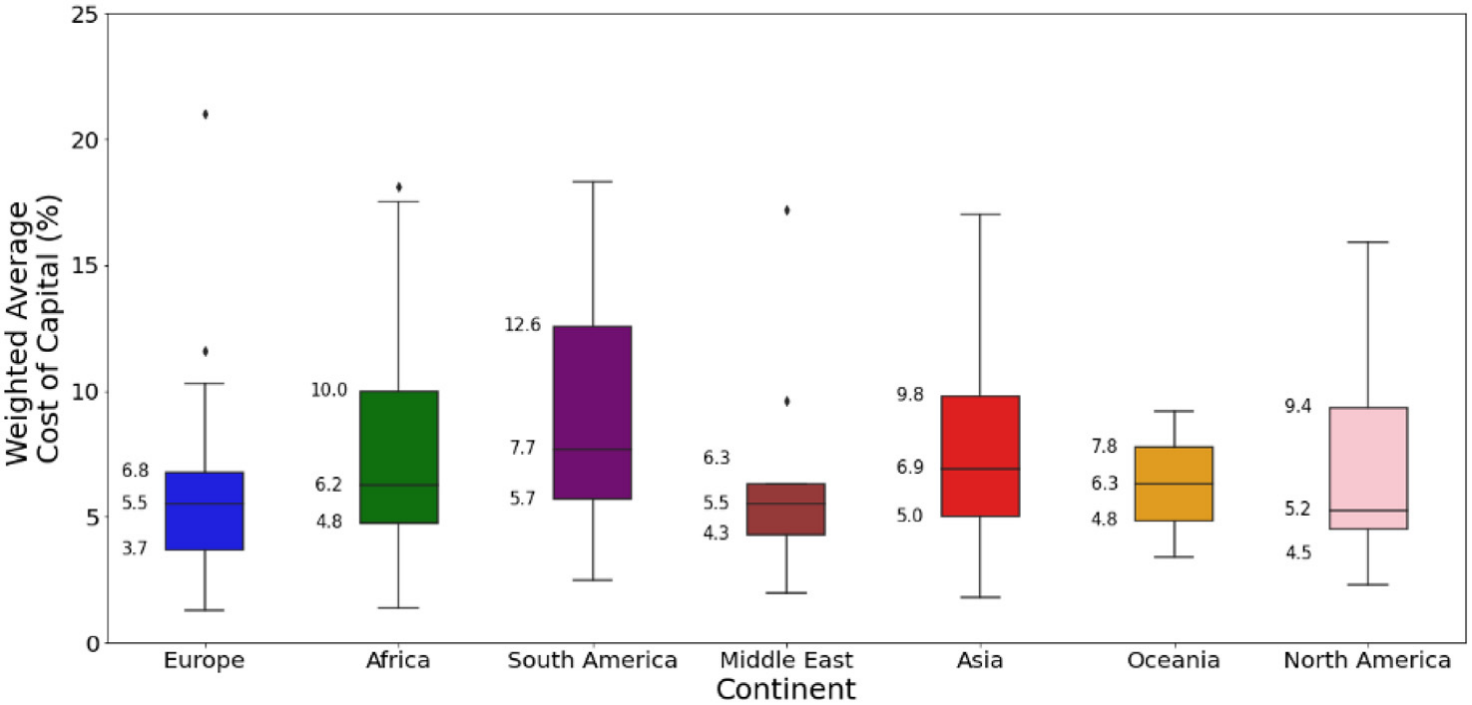
Range of financing costs for solar farms in each world region, where available, in 2020 to 2024.

Figs. 9–11 present an excerpt of the values exhibited across the literature for key parameters for the recent period (2020 to 2024), alongside projections for the near future (2030) and far future (2050). Fig. 9 presents the range of capital costs and fixed operating costs for the different categories of solar farms for 2020 to 2024 while Fig. 10, Fig. 11 present values of capital costs and financing costs for large-scale fixed solar PV (the aggregate of ‘Fixed Axis’ and ‘Unspecified’ categories).

The median values for the recent period (2020 to 2024), aggregated across all world regions and all technology sub-types was 975 USD/kW for capex, 21.8 USD/kW/yr for fixed O&M, 13.9 USD/MWh for total O&M, 2 years for construction time, 30 years for operating life and 6 % for cost of capital. There were no data on variable O&M, although this is typically considered to be zero.

### 3.8 Summary of values for wind power

Table 8 compiles the sources used for each variable and how many values were collated.

**Table 8:** The number of datapoints collected and sources used for each parameter in the database, for wind power.

Parameter	Number of datapoints	Sources
Capital cost	915	[[3], [4], [5], [6], [7], [8],^10^,^11^,[15], [16], [17], [18], [19],[25], [26], [27], [28],^35^,[45], [46], [47], [48]]
Variable operating costs	10	[10,28]
Fixed operating costs	211	[[5], [6], [7],^10^,^11^,^15^,[26], [27], [28]]
Total operating costs	140	[4,5,[17], [18], [19],^23^,^35^,[45], [46], [47], [48]]
Fuel price	Not used	
Cost of capital	915	[10,25,41,45,49,50]
Efficiency	Not used	
Construction time	36	[5,6,10,11,17,18,26,28,51]
Operational lifetime	52	[3,6,7,10,11,15,19,[26], [27], [28]]

**Fig. 12:**
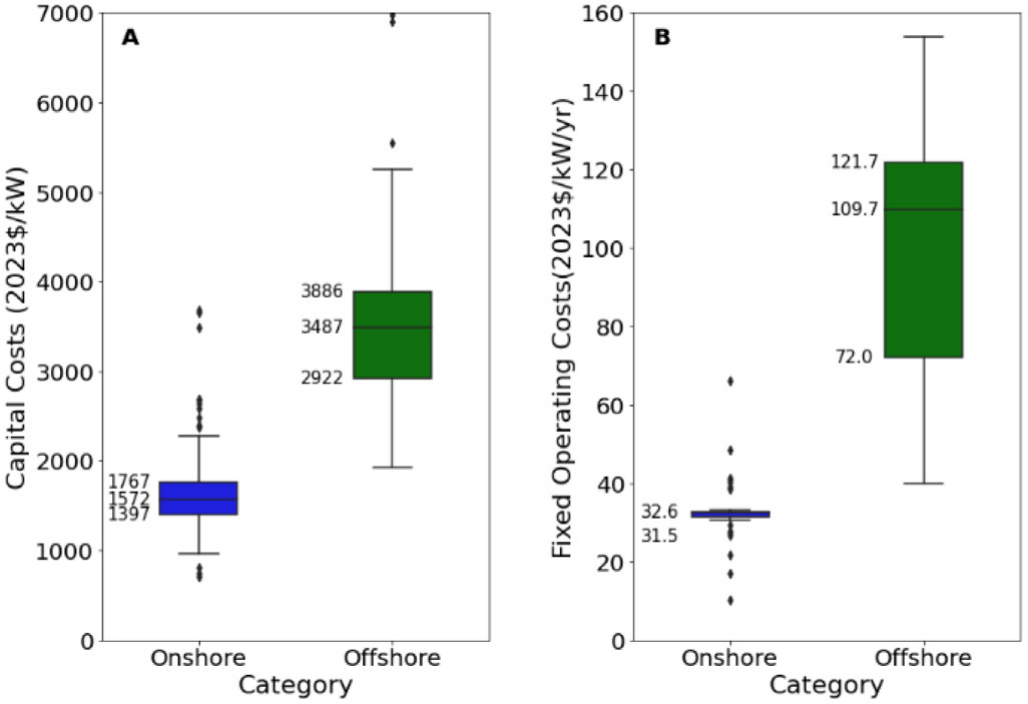
Range of a) capital costs and b) fixed operating costs, for onshore and offshore wind farms in 2020 to 2024.

**Fig. 13:**
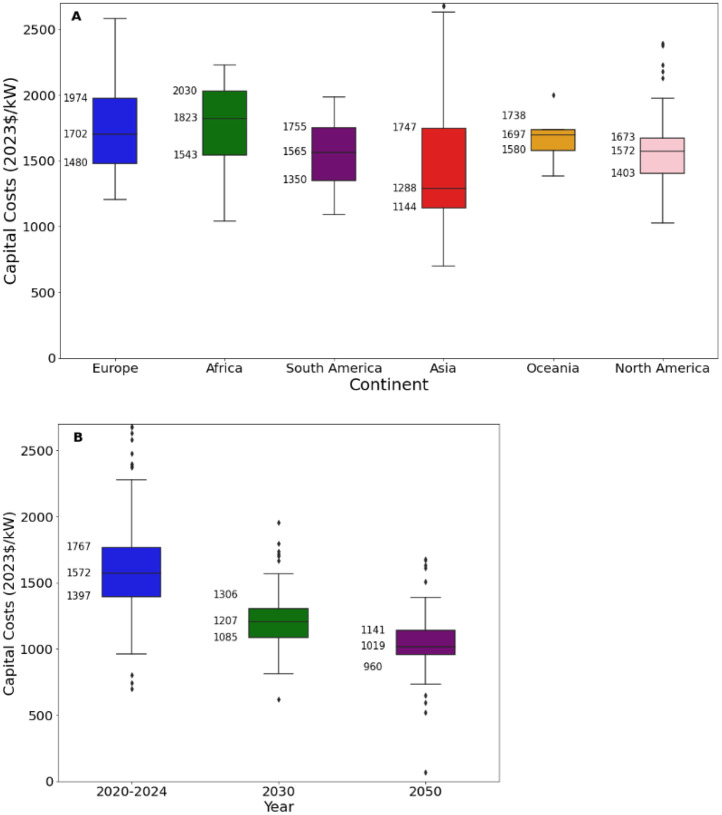
Range of capital costs for onshore wind farms in a) 2020 to 2024 for each world region, where available, and b) global projections for 2030 and 2050.

**Fig. 14:**
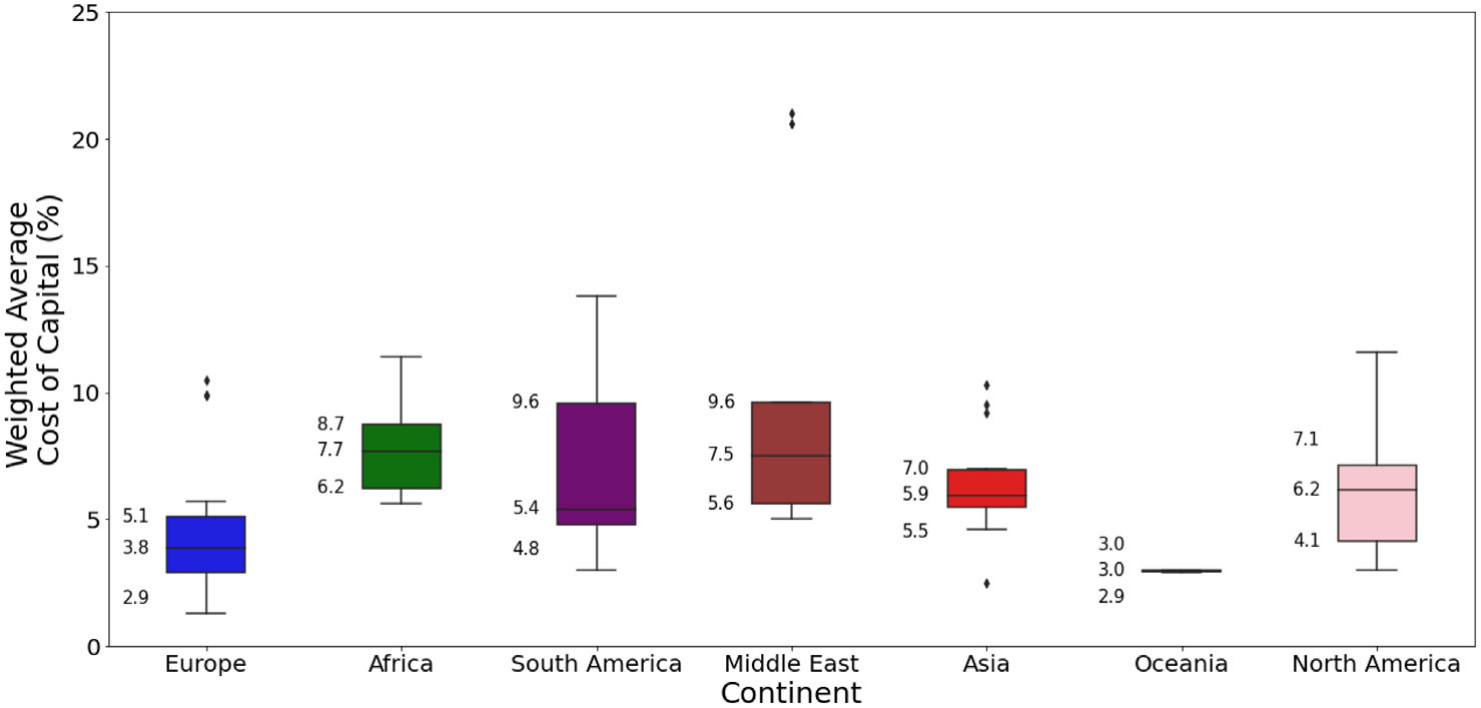
Range of financing costs for onshore wind farms in each world region, where available, in 2020 to 2024.

Figs. 12–14 present an excerpt of the values exhibited across the literature for key parameters for the recent period (2020 to 2024), alongside projections for the near future (2030) and far future (2050). Fig. 12 presents the range of fixed operating and capital costs for onshore and offshore wind farms for 2020 to 2024 while Fig. 13, Fig. 14 present values of capital costs and financing costs for onshore wind farms.

The median values for the recent period (2020 to 2024), aggregated across all world regions and all technology sub-types was 1750 USD/kW for capex, 32.6 USD/kW/yr for fixed O&M, 3.6 USD/MWh for variable O&M, 30.9 USD/MWh for total O&M, 3 years for construction time, 25 years for operating life and 5.6 % for cost of capital.

### 3.9 Summary of values for batteries

Table 9 compiles the sources used for each variable and how many values were collated.

**Table 9:** The number of datapoints collected and sources used for each parameter in the database for BESS.

Parameter	Number of datapoints	Sources
Capital cost	333	[3,6,7,11,13,17,18,26,[52], [53], [54], [55], [56], [57], [58], [59]]
Variable operating costs	17	[13,54,57,58]
Fixed operating costs	182	[6,11,13,17,18,26,36,54,55,57,58]
Total operating costs	No data found	
Fuel price	Not used	
Cost of capital	No data found	
Efficiency	60	[7,11,13,54,55,[57], [58], [59], [60], [61]]
Construction time	25	[6,13,17,26,55,60]
Operational lifetime	54	[3,6,7,11,26,54,55,57,[59], [60], [61]]

**Fig. 15:**
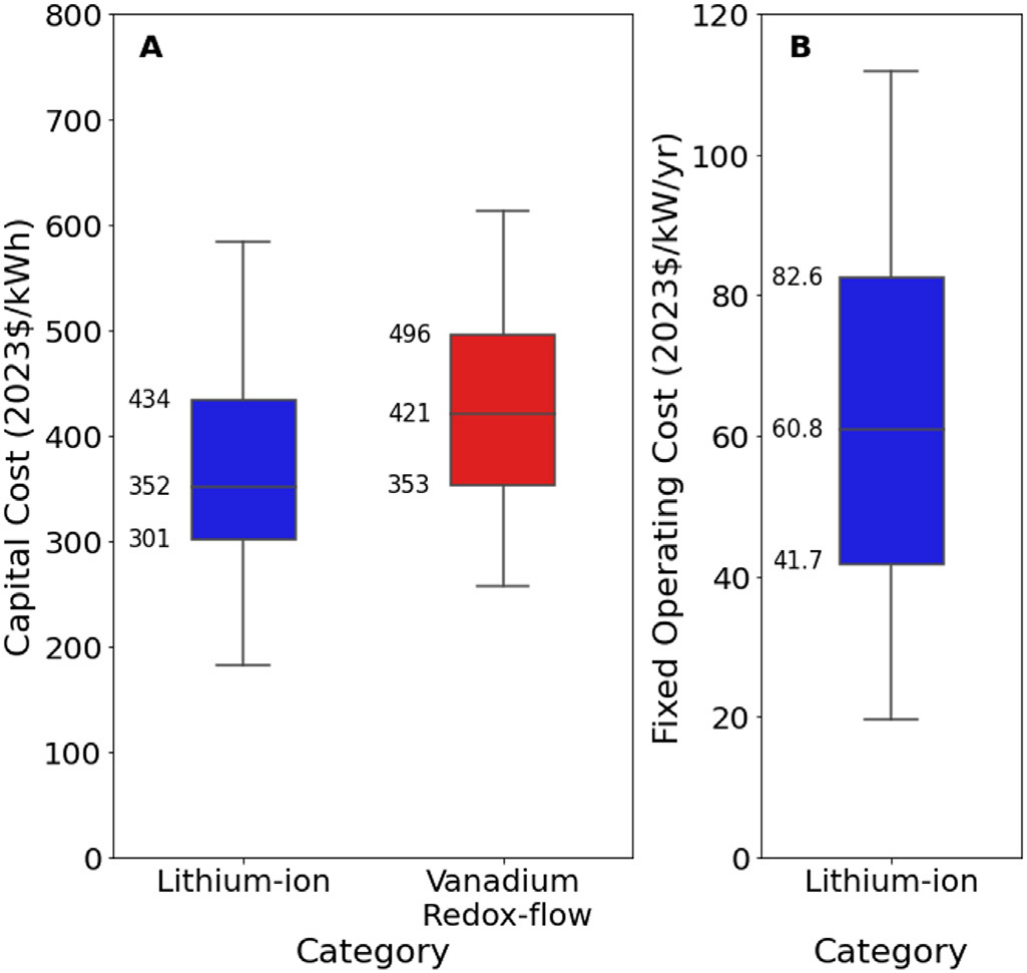
Range of a) capital costs and b) fixed operating costs in 2020 to 2024, for lithium-ion and vanadium redox-flow battery energy storage systems. There was no data available on fixed operating costs for vanadium redox-flow batteries between 2020 and 2024.

**Fig. 16:**
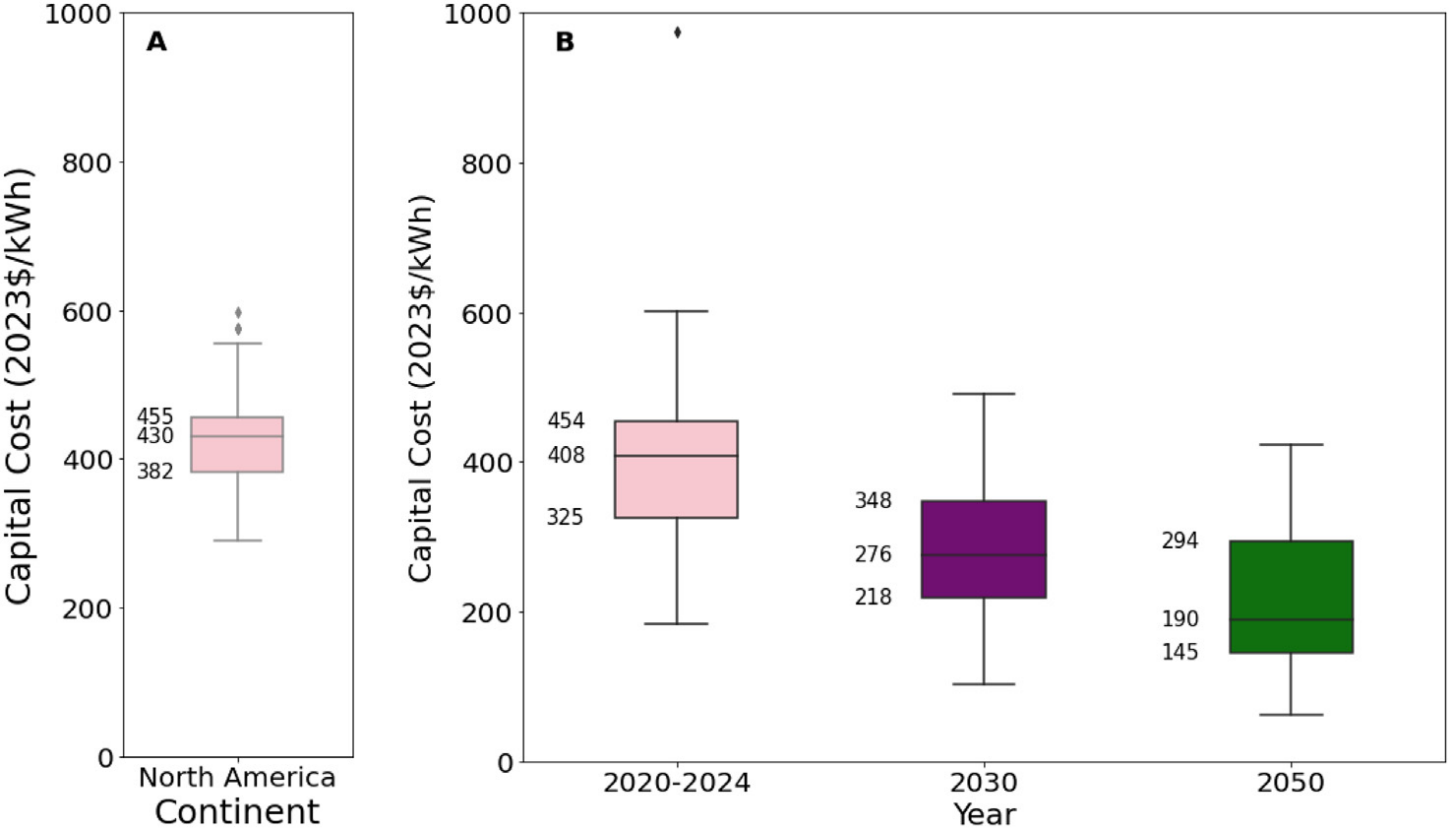
Range of capital costs for lithium-ion battery energy storage systems in a) 2020 to 2024 for each world region, where available, and b) global projections for 2030 and 2050.

Fig. 15, Fig. 16 present an excerpt of the values exhibited across the literature for key parameters for the recent period (2020 to 2024), alongside projections for the near future (2030) and far future (2050). Fig. 15 presents the range of fixed operating and capital costs for lithium-ion batteries (an aggregate of the ‘Lithium-ion’, ‘Lithium-ion NMC’ and ‘Lithium-ion LFP’ categories) for 2020 to 2024, whilst Fig. 16 presents values of capital costs for lithium-ion battery energy storage systems, both regional and projected.

The median values for the recent period (2020 to 2024), aggregated across all world regions and all technology sub-types was 402 USD/kWh for capex, 60.8 USD/kW/yr for fixed O&M, 0.6 USD/MWh for variable O&M, 85 % for efficiency, 1 years for construction time, and 15 years for operating life [6,11,54,59].

### 3.10 Demonstration of calculating LCOE

To give an example application, the GNESTE database can be used to calculate the Levelized Cost of Electricity (LCOE), a widely-used metric for comparing the economic efficiency of different generating technologies. Adapting the IEA's formula [3] to use our variable names, LCOE can be calculated via:

(1) $$LCOE=\;\frac{\sum_{t}\left(CAPEX_{t}+OPEX_{t}+FUEL_{t}+CARBON_{t}\right)\cdot {\left(1+WACC\right)}^{-t}\;}{\sum_{t}ENERGY_{t}\cdot {\left(1+WACC\right)}^{-t}}$$

Where *t* is the year, and both the numerator and denominator sums run from *t* = 0 to *LIFETIME*. The denominator contains the specific energy output (in MWh per kW capacity):

(2) ENERGY=8.76·CF

where *CF* is the capacity factor, or utilization of the technology, which is user-defined based on the specific application, location and market. Total operations & maintenance cost (in $/kW/yr) is given by the *OPEX_T* variable, or can be calculated as:

(3) OPEX=OPEX_F+OPEX_V·ENERGY

The annual cost of fuel input (in $/kW/yr) is given by:

(4) $$FUEL=\frac{FUEL\_PRICE}{EFFICIENCY}\cdot ENERGY$$

And finally, if relevant, the annual cost of carbon emissions (in $/kW/yr) is given by:

(5) $$CARBON=\frac{FUEL\_CI}{EFFICIENCY}\cdot CARBON_{PRICE}\cdot ENERGY$$

Where *FUEL_CI* is 344.5 kgCO_2_/MWh for coal [62] or 364.2 kgCO_2_/MWh for lignite [63], 204.8 kgCO_2_/MWh for natural gas [62], and zero for hydro, nuclear, solar, and wind. *CARBON_PRICE* is the cost of emitting a tonne of CO_2_, which is user-defined based on the market and scenario considered.

To give a simple demonstration of how the GNESTE database can be used for techno-economic calculations, Eqs. (6)–(11) calculate the LCOE of each electricity generating technology in turn. Each calculation is based on the median values from the GNESTE database listed in the previous sections, assuming all capital expense is occurred in a single year (at the start of the project), all other variables remain constant (in real terms) over the project lifetime, and there is no price on carbon emissions (*CARBON_PRICE* = 0 USD/tCO_2_). These examples could be made more accurate by including details such as the build time (meaning energy production does not occur until later and thus is more discounted), interest during construction, output declining with age, or decommissioning cost.

For coal-fired power generation (without CCS):

(6) $$LCOE=\;\frac{2000+\sum_{t}\left(74.66+108.98+0\right)\cdot {\left(1+0.10\right)}^{-t}\;}{\sum_{t}7.45\cdot {\left(1+0.10\right)}^{-t}}=49.58\frac{USD}{MWh}$$

Using example values of *CF* = 85 %, *WACC* = 10 %, and *EFFICIENCY* = 44 % (the median for non-CCS coal plants). This compares to Lazard's estimate of 68 USD/MWh for new-build coal (low-case, excluding CCS) [11], which is notably higher as Lazard estimates capex to be 60 % higher than found here.

For combined-cycle gas power generation (without CCS):

(7) $$LCOE=\;\frac{1060+\sum_{t}\left(47.84+161.79+0\right)\cdot {\left(1+0.10\right)}^{-t}\;}{\sum_{t}7.88\cdot {\left(1+0.10\right)}^{-t}}=40.19\frac{USD}{MWh}\;$$

using example values of *CF* = 90 %, WACC = 10 %, and *EFFICIENCY* = 57.5 % (the median for unabated CCGT plants). This compares to Lazard's estimate of 39 USD/MWh for new-build combined-cycle gas (low-case, no carbon pricing, excluding CCS) [11].

For hydroelectric power generation (averaged across all types):

(8) $$LCOE=\;\frac{4491+\sum_{t}\left(94.05+0+0\right)\cdot {\left(1+0.10\right)}^{-t}\;}{\sum_{t}4.38\cdot {\left(1+0.10\right)}^{-t}}=115.41\frac{USD}{MWh}\;$$

using example values of *CF* = 50 % and *WACC* = 10 %.

For nuclear power (averaged across all types):

(9) $$LCOE=\;\frac{7350+\sum_{t}\left(155.9+143.16+0\right)\cdot {\left(1+0.10\right)}^{-t}\;}{\sum_{t}7.88\cdot {\left(1+0.10\right)}^{-t}}=124.43\frac{USD}{MWh}\;$$

using example values of *CF* = 90 % and *WACC* = 10 %. This compares to Lazard's estimate of 141–221 USD/MWh for new-build nuclear [11], which is notably higher as Lazard estimates capex to be 15–85 % higher than found here.

For fixed-axis solar PV:

(10) $$LCOE=\;\frac{954+\sum_{t}\left(19.7+0+0\right)\cdot {\left(1+0.06\right)}^{-t}\;}{\sum_{t}1.31\cdot {\left(1+0.06\right)}^{-t}}=64.75\frac{USD}{MWh}\;$$

using an example value of *CF* = 15 %, and *CAPEX* = 954 USD/kW (the median of ‘Fixed Axis’ and ‘Unspecified’ categories). This compares to Lazard's high-case estimate of 96 USD/MWh for utility-scale solar PV, and low-case estimate of 49 USD/MWh for commercial and industrial solar PV [11].

For onshore wind:

(11) $$LCOE=\;\frac{1572+\sum_{t}\left(42.06+0+0\right)\cdot {\left(1+0.056\right)}^{-t}\;}{\sum_{t}2.63\cdot {\left(1+0.056\right)}^{-t}}=57.88\frac{USD}{MWh}\;$$

using an example value of *CF* = 30 %. This compares to Lazard's estimates which range from 24 to 75 USD/MWh for onshore wind [11].

### 3.11 Demonstration of calculating LCOS

For battery storage, the GNESTE database can instead be used to calculate the Levelized Cost of Storage (LCOS), a widely-used metric for comparing the economic efficiency of different storage technologies. Adapting Schmidt's formula [13] to use our variable names, LCOS can be calculated via:

(12) $$LCOS=\;\frac{\sum_{t}\left(CAPEX_{t}+OPEX_{t}+CHARGING_{t}\right)\cdot {\left(1+WACC\right)}^{-t}\;}{\sum_{t}ENERGY_{t}\cdot {\left(1+WACC\right)}^{-t}}$$

Where the cost of charging the battery (in $/kW/yr) is given by:

(13) $$CHARGING=\frac{ELECTRICITY\_PRICE}{EFFICIENCY}\cdot ENERGY$$

And *ELECTRICITY_PRICE* is the volume-weighted average price for electricity used to charge the storage system, which is user-defined based on the market and scenario considered.

For lithium-ion energy storage systems with 4-h duration:

(14) $$LCOS=\;\frac{1692+\sum_{t}\left(58.62+82.06+0\right)\cdot {\left(1+0.10\right)}^{-t}\;}{\sum_{t}1.4\cdot {\left(1+0.10\right)}^{-t}}=235.81\frac{USD}{MWh}\;$$

Using example values of *CAPEX* = (423 USD/kWh (the median for the ‘lithium-ion’ category) × 4 h = 1608 USD/kW), *ENERGY* = (4 h/cycle × 350 cycles/yr) = 1400 MWh/yr per MW capacity, *ELECTRICITY_PRICE* = 50 USD/MWh (which gives a fuel price of 61.22 USD/kWh given the median lithium-ion round-trip efficiency of 85.3 %), and *WACC* = 10 %. This compares to Lazard's estimate of 200–257 USD/MWh for 4-h duration utility-scale lithium-ion storage [11].

## 4 Experimental Design, Materials and Methods

The data set was collated by reviewing reports, websites, and datasets from international and national organisations, and peer-reviewed journal papers. The search for additional sources was conducted until we reached a saturation of information. Only primary sources were used, and care was taken to ensure that data points were not duplicated across sources. Sources were prioritised according to the robustness of their methodologies and representativeness of data. For example, surveys of actual installed costs were prioritised over modelled estimates. All data entries were reviewed by each member of the research team for quality assurance. Raw data were converted to the standardised units shown in Table 1, with all currencies converted to 2023 US Dollars using the source country's GDP deflator as a measure of general inflation to convert into 2023 local currency [64], then using the 2023 year-average market exchange rate to convert to US Dollars [65]. All currency conversion rates are listed within the metadata that accompanies the database.

## Limitations

Data were more widely available for OECD nations, particularly in Europe and North America, and the larger BRICS countries, notably India and China, which are the focus of many assessments. Substantial data gaps exist among primary sources, and thus in our database, for Africa, South America, and some areas of Asia, for all technologies included in the GNESTE database. Data on the cost of capital is scarce, except for wind and solar. There was limited data for many categories of technologies, e.g., most data for batteries were available for lithium-ion batteries. Projected data values are based on various modelling techniques and not measured data. Future values are speculative and uncertain as cost and performance vary as a function of deployment (according to technological learning rates), policies, and market dynamics among other factors.

For coal and gas power, the database does not include data on the emissions intensity of electricity generation, but this can be calculated by combining the efficiencies from the database with fuel carbon content. Likewise, the database does not detail the installed capacities of technologies or how much electricity they produce as this is readily available from many open sources. It also excludes data on capacity factors as these are a function of the market plants operate in, required utilisation and competition with other plants which are highly context-specific. The database does not include values for levelized cost of electricity (LCOE), as these are also highly context-specific. Instead, it provides the essential inputs for researchers to calculate this themselves using parameters from the database as demonstrated above.

## Ethics Statement

The authors have read and followed the ethical requirements for publication and confirm that this work does not involve human subjects, animal experiments, or any data collected from social media platforms.

## CRediT authorship contribution statement

**Luke Hatton:** Formal analysis, Data curation, Visualization, Writing – original draft, Writing – review & editing. **Nathan Johnson:** Formal analysis, Data curation, Writing – original draft, Writing – review & editing. **Lara Dixon:** Data curation, Writing – review & editing. **Bosi Mosongo:** Writing – review & editing. **Savanha De Kock:** Writing – review & editing. **Andrew Marquard:** Writing – review & editing. **Mark Howells:** Conceptualization, Supervision, Funding acquisition. **Iain Staffell:** Formal analysis, Data curation, Writing – original draft, Writing – review & editing.

## Data Availability

The Global and National Energy Systems Techno-Economic (GNESTE) Database: Cost and performance data for electricity generation and storage technologies (Original data) (Zenodo).
